# Supplementary material for: Maqui Berry and Ginseng Extracts Reduce Cigarette Smoke-Induced Cell Injury in a 3D Bone Co-Culture Model
Source: Antioxidants (Basel). 2022 Dec 14;11(12):2460. doi: 10.3390/antiox11122460 (PMC9774157; doi:10.3390/antiox11122460)
Supplement: Supplementary file 1 [file antioxidants-11-02460-s001.zip › antioxidants-1982676-supplementary.pdf]

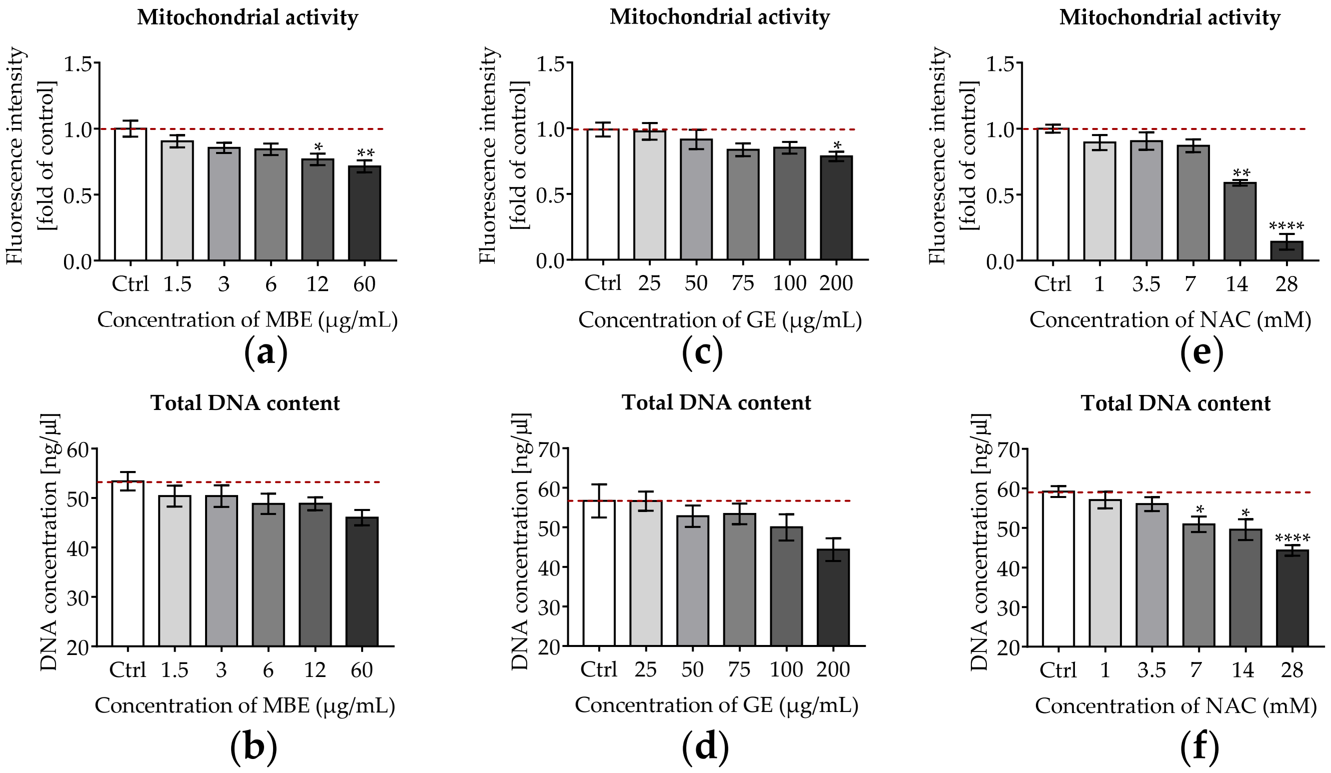

Supplementary Figure S1. Cytotoxicity tests of MBE, GE, and NAC on SCP-1/THP-1 co-culture system. The mitochondrial activity and total DNA content were measured on day 14 to determine the cell viability and cell number in the co-culture system. Mitochondrial activity of the co-culture system with the intervention with MBE(a), GE (e), and NAC (e). Total DNA content of the co-culture system after being exposed to different concentrations of MBE (b), GE (d), and NAC (f). Statistical differences were determined using the Kruskal-Wallis test followed by Dunn's multiple comparison test. Data are present as mean± SEM, and the significance was shown as * $p<0.05$, ** $p<0.01$, and **** $p<0.001$ vs Ctrl group. N = 3, n = 3.

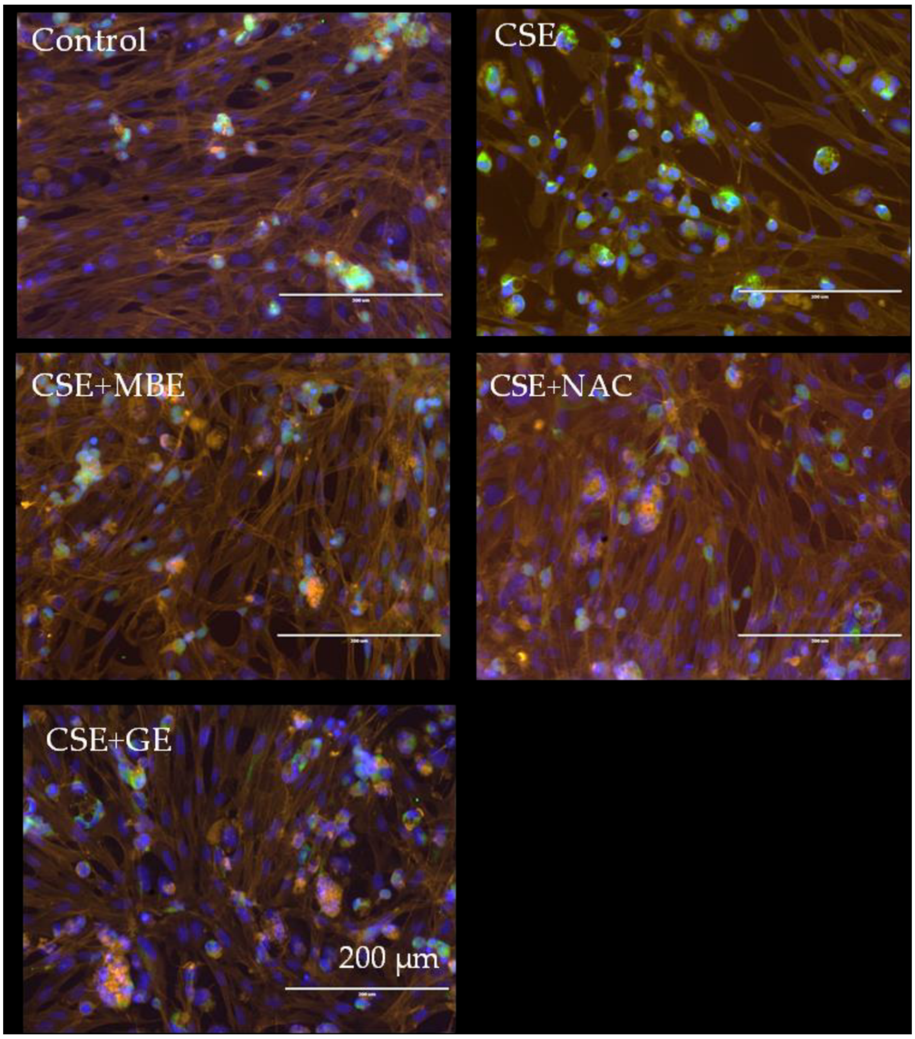

Supplementary Figure S2. Representative images of SCP-1 and THP-1 cells coculture at different conditions (control, CSE, CSE+MBE, CSE+NAC, and CSE+GE), stained for actin (phalloidin-TRITC; red) , nucleus (Hoechst 33342; blue) and NF-κB intensity (green) on day 7 (scale bar 200 µm). Mononuclear cells positive for actin filaments were considered osteoblast-like cells (SCP-1 cells). Polynuclear cells, positive for an actin ring structure were considered osteoclast-like cells (THP-1 cells).
